# The buffy coat method: a tool for detection of blood parasites without staining procedures

**DOI:** 10.1186/s13071-020-3984-8

**Published:** 2020-02-27

**Authors:** Carolina R. F. Chagas, Rasa Binkienė, Mikas Ilgūnas, Tatjana Iezhova, Gediminas Valkiūnas

**Affiliations:** Nature Research Centre, Akademijos 2, 08412 Vilnius, Lithuania

**Keywords:** Blood parasites, Parasite diagnostics, Birds, *Haemoproteus*, *Plasmodium*, *Leucocytozoon*, *Trypanosoma*, *Lankesterella*, Microfilaria, Fieldwork

## Abstract

**Background:**

Blood parasites belonging to the Apicomplexa, Trypanosomatidae and Filarioidea are widespread in birds and have been studied extensively. Microscopical examination (ME) of stained blood films remains the gold standard method for the detection of these infections in birds, particularly because co-infections predominate in wildlife. None of the available molecular tools can detect all co-infections at the same time, but ME provides opportunities for this to be achieved. However, fixation, drying and staining of blood films as well as their ME are relatively time-consuming. This limits the detection of infected hosts during fieldwork when captured animals should be released soon after sampling. It is an obstacle for quick selection of donor hosts for parasite experimental, histological and other investigations in the field. This study modified, tested and described the buffy coat method (BCM) for quick diagnostics (~ 20 min/sample) of avian blood parasites.

**Methods:**

Blood of 345 birds belonging to 42 species was collected, and each sample was examined using ME of stained blood films and the buffy coat, which was examined after centrifugation in capillary tubes and after being transferred to objective glass slides. Parasite detection using these methods was compared using sensitivity, specificity, positive and negative predictive values and Cohen’s kappa index.

**Results:**

*Haemoproteus*, *Leucocytozoon*, *Plasmodium*, microfilariae, *Trypanosoma* and *Lankesterella* parasites were detected. BCM had a high sensitivity (> 90%) and specificity (> 90%) for detection of *Haemoproteus* and microfilariae infections. It was of moderate sensitivity (57%) and high specificity (> 90%) for *Lankesterella* infections, but of low sensitivity (20%) and high specificity (> 90%) for *Leucocytozoon* infections. *Trypanosoma* and *Plasmodium* parasites were detected only by BCM and ME, respectively. According to Cohen’s kappa index, the agreement between two diagnostic tools was substantial for *Haemoproteus* (0.80), moderate for *Lankesterella* (0.46) and fair for microfilariae and *Leucocytozoon* (0.28) infections.

**Conclusions:**

BCM is sensitive and recommended as a quick and reliable tool to detect *Haemoproteus*, *Trypanosoma* and microfilariae parasites during fieldwork. However, it is not suitable for detection of species of *Leucocytozoon* and *Plasmodium*. BCM is a useful tool for diagnostics of blood parasite co-infections. Its application might be extended to studies of blood parasites in other vertebrates during field studies.
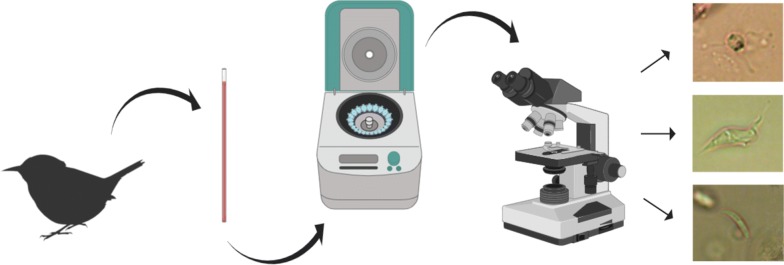

## Background

Blood parasites belonging to the Apicomplexa, Trypanosomatidae, and microfilariae of filariid nematodes (Filarioidea) are widespread and have been extensively reported in different bird groups all over the world [1–10]. Blood parasites also infect fishes [11, 12], amphibians [13, 14], reptiles [15] and mammals [16]. Nowadays, there are two main methods, which are broadly used to diagnose these infections: microscopic examination (ME) of blood films, usually stained with Giemsa; and polymerase chain reaction (PCR)-based testing [13, 17–29].

ME is useful to detect infections not only at relatively high levels of parasitemia, but also during light chronic infections [19]. It is also recommended for detection of co-infections, which predominate in wildlife [19, 30, 31]. Available PCR-based diagnostic protocols using general primers are often insensitive to detect co-infections of closely related blood parasites in many taxonomic groups [30–33]. Compared to the molecular protocols, blood film ME is cheaper, often faster and can be used even during fieldwork if access to relatively simple microscopic facilities is available. Additionally, molecular characterization of the majority of described blood parasite species remains to be developed; however, many of the species or genera can be distinguished using morphological characters of the blood stages. From this point of view, ME of stained blood films can still be considered as the gold standard method for blood parasite biodiversity research in wildlife. However, this methodology is rather time-consuming for field studies, and this creates obstacles in examining large numbers of animals at the study site in the wild. Additionally, it requires good quality blood films, which must be fixed, dried and stained properly to reach good results during microscopic diagnostics [19]. Proper staining and the subsequent ME procedures are often difficult to achieve during fieldwork, particularly in remote areas.

Several studies reported that PCR could potentially be more sensitive for detection of avian haemosporidian parasites, especially during light chronic infections [17, 18]. However, professional microscopic examination can also be just as sensitive in detection of blood parasites. It is important to note that PCR-based techniques provide other valuable data in addition to simple parasite detection, particularly regarding parasite DNA sequence information, which can be used for populational genetics, phylogenetics, epidemiology, vector and other studies [10, 22, 23, 34–38]. Unfortunately, the application of PCR-based techniques is usually impossible during fieldwork due to strict requirements for materials and laboratory infrastructure. Moreover, molecular diagnostic procedures (DNA extraction, PCR, electrophoresis, DNA precipitation and sequencing) are also time-consuming, and depending on the PCR-based protocols applied, it usually takes several days to obtain sequence information.

Quick detection of infected animals in the wild is essential for collection of parasite donor-hosts, which are often needed for helminthology [39], experimental parasitology [40, 41], parasite identification [31, 35, 42] and host pathology [43, 44] research. This is a particularly sensitive issue aimed at minimizing the harm to wildlife, when only a few parasitized individuals could be caged or euthanized if essentially necessary for collection of pathogens, and all other sampled animals can be quickly released according to permit requirements at study sites. In order to optimize and facilitate the diagnostics of blood parasites, a concentration technique was developed for application in parasitology research, the buffy coat method (BCM). The method is based on blood centrifugation and the resulting separation of blood cells and parasites in different layers [45]. It has been commonly used in parasitology, particularly for detection of *Trypanosoma* species and microfilariae in humans [46–48] and domestic animals [49–52]. A similar technique was also used to diagnose apicomplexan parasites in humans such as *Babesia* spp. [53] and *Plasmodium* spp. [54–57]. However, there are few reports on the application of BCM in detection of parasites in wild birds [9, 58–61]. The first application of BCM for detection of bird blood parasites was described by Bennett [58]. Yet, according to his protocol, it was necessary to stain the preparations after blood centrifugation in order to distinguish the parasites. Bennett [58] recognised that he was able to detect *Haemoproteus* and *Leucocytozoon* infections in wild birds using BCM, but this technique has not been applied broadly for detection of avian haemosporidian parasites.

The main goals of this study were: (i) to modify the BCM for more practical use in avian blood parasite sampling during fieldwork; (ii) to test the sensitivity and specificity of the BCM in detection of blood parasites in naturally infected birds using non-stained preparations; (iii) to evaluate the effectiveness of ME of the non-stained preparations collected using BCM in comparison to the ME of Giemsa stained blood films.

## Methods

### Field work and sample collection

Blood samples were collected from 345 birds of 42 species belonging to 20 families and 2 orders (Additional file 1: Table S1) in Ventės Ragas Ornithological Station, Lithuania (55°20′28.1″N, 21°11′25.3″E) during May 2019. Birds were caught using “Rybachy” type funnel traps, mist nets and “Zigzag” traps. Approximately 50 µl of blood was taken by puncturing the brachial vein and collected using heparinized capillary tubes. A few drops of blood were used to prepare blood films, and the remaining blood was maintained in the capillary tubes to be examined using BCM.

Blood films were air-dried using a battery-powered fan, fixed by immersion in absolute methanol for 1–2 s, dried at room temperature using a fan and stained using a fast protocol (blood films were stained in 30% Giemsa solution for 15 min at temperature of ~ 20 °C) [62]. This protocol provided good results in distinguishing parasites in blood stages.

### Microscopical examination

One stained blood film from each bird was examined using an Olympus CX23 light microscope (Olympus, Tokyo, Japan) by experienced parasitologists. Approximately 10,000 red blood cells were screened at high magnification (1000×) in each blood film. Quick staining and ME of blood films is essential as it provides an opportunity for quick examination and therefore the subsequent release of birds that are non-infected or are non-suitable for further studies, minimizing any potential suffering of sampled individuals. Images of reported parasites were prepared using a Zeiss PrimoStar light microscope equipped with an Axiomcam ERc 5s camera (Carl Zeiss MicroImaging GmbH, Jena, Germany).

### Buffy coat method

**Fig. 1:**
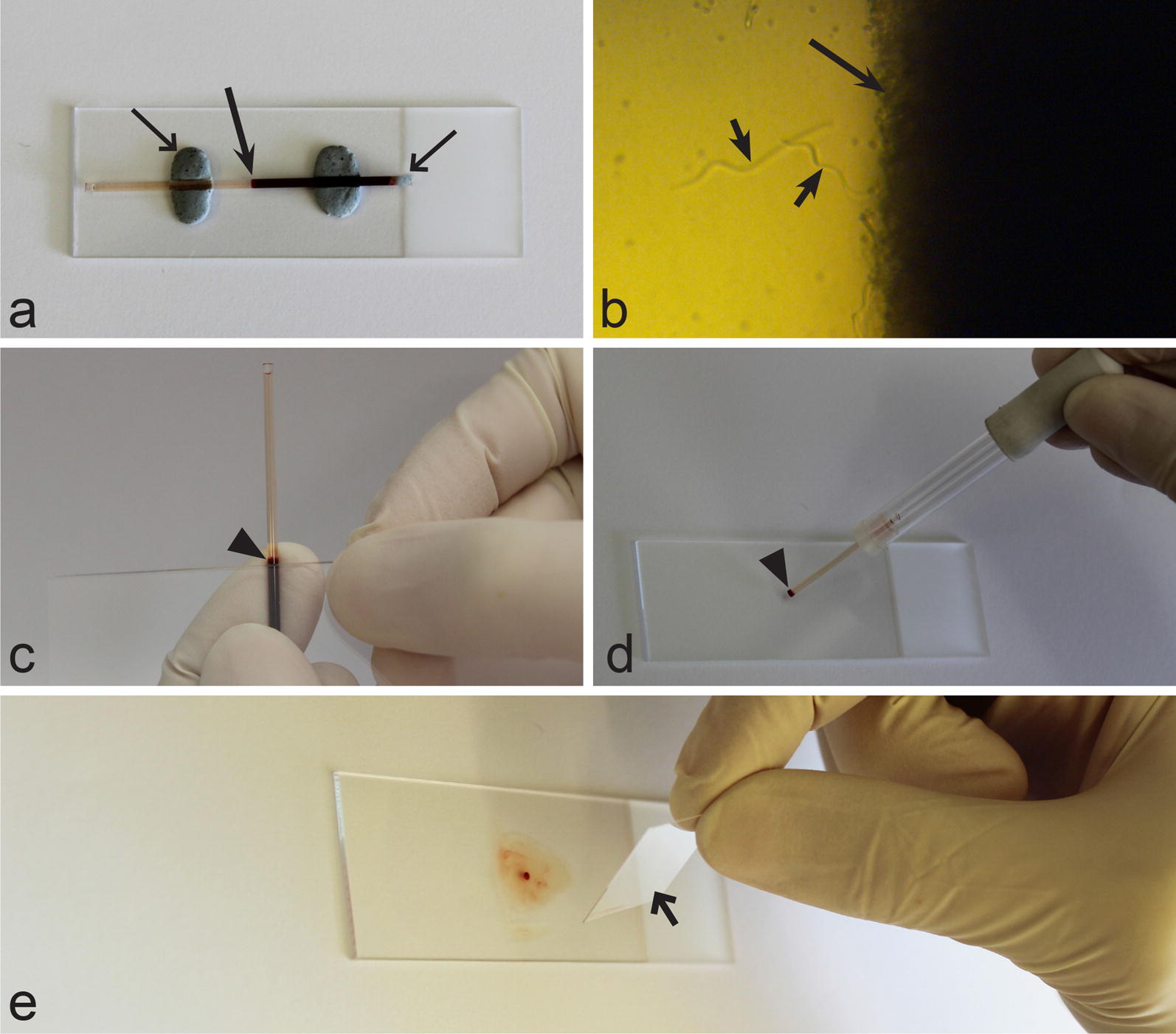
Main procedures of blood sample preparation for the application of buffy coat method. **a** Capillary tube with centrifugated blood, which was prepared for initial microscopical examination (note that one tip of the capillary is blocked with plasticine and the entire capillary tube is fixed on the objective glass slide using plasticine). Long barbed arrow, buffy coat; long simple arrow, plasticine. **b** Buffy coat layer as it looks under light microscope (100× magnification), note that microfilariae are readily visible and locate close to the buffy coat layer (long arrow, buffy coat layer; short arrows, microfilariae). **c** Capillary tube being broken close to the buffy coat layer using a sharp edge of the objective glass slide (arrowhead, the site where the capillary tube should be broken). **d** Blood and the buffy coat layer being transferred to the objective glass slide (arrowhead, buffy coat layer and small portion of red blood cells). **e** Blood and the buffy coat layer transferred on the objective glass slide and being covered with a coverslip (arrow, cover slip)

Each blood sample was examined using the BCM in parallel to ME. The heparinized capillary tubes with blood were sealed with plasticine at one end (Fig. 1a) and then centrifugated in a microhematocrit centrifuge for 5 min at 10,000× *rpm*. Centrifugation was performed 5–10 min after withdrawal of blood from the birds. Next, the capillary tube was placed above a glass slide fixed with plasticine, the buffy coat area (Fig. 1a) was then examined under low magnification (100×) that was effective for visualization of motile stages of relatively large parasites (microfilariae and *Trypanosoma* spp.) (Fig. 1b). Then, the buffy coat wet preparations were prepared. The capillary tube was broken at the site of the red blood cell layer, approximately 1 mm below the buffy coat layer (Fig. 1c). This was achieved by gently pressing and running the sharp edge of an objective glass slide on the glass capillary tube. The buffy coat with the adjacent plasma were transferred to an objective glass slide using a capillary tube pump (Fig. 1d), followed by gentle mixing and covering with a coverslip (size of 18 × 18 mm) (Fig. 1e). Finally, this wet preparation was left for approximately 1 min to allow the blood cells to settle on the slide, then the entire preparation was examined for the presence of parasites under microscope at 400× magnification. Parasite images were prepared using a Zeiss PrimoStar light microscope equipped with Axiomcam ERc 5s camera (Carl Zeiss). After the ME, the coverslip was removed and a thin film was prepared on the objective glass slide, the film was dried, fixed, stained and examined as a blood film.

It is important to note that care should be taken while applying the BCM to guarantee reliable results, especially when many samples are being processed in parallel. It is mandatory that the samples be examined microscopically immediately after centrifugation, otherwise the actively moving parasites (particularly microfilariae and large trypanosomes) can merge in the red blood cell layer or move into the plasma [46], resulting in possible false negative results. Similar care should also be taken with the wet preparations as they can also quickly dry out on the objective glass slides after preparation. It is therefore recommended to keep the preparations in a humid chamber until examination, especially when multiple samples need to be checked in parallel. Buffy coat wet preparations should be carefully prepared in a way to avoid large volumes of red blood cells being transferred to the slides; this preparation should be thin. If many red blood cells are in these preparations, the cells could form clusters, which prevent the observer from properly identifying or detecting parasites present in the samples. It is also necessary to prepare the buffy coat wet preparations carefully in order to avoid any small pieces of glass that might appear in the preparations, which can result from when the glass capillary is broken; such artefacts might prevent an even preparation of blood films and compromise microscopical examination.

### Statistical analysis

Statistical analysis was carried out using the Statistical Package for the Social Sciences (SPSS) for Windows, version 15.0 (SPSS Inc., Chicago, IL, USA). For comparison with BCM, ME was considered as the gold standard method during the statistical analysis. Parasite detection results were compared between these two diagnostic methods, and the sensitivity (probability to get positive results when the infection is present), the specificity (probability to get negative results when the infection is absent), the positive predictive value (proportion of individuals with positive test results that actually have the infection) and the negative predictive value (proportion of individuals with negative test results that actually do not have the infection) were calculated [63], along with their respective 95% confidence interval (95% CI). A *P*-value of ≤ 0.05 was considered as significant. Cohen’s kappa index (κ) and its 95% CI were also calculated to evaluate concordances between BCM and ME methods [64]. The concordance was considered according to Landis & Koch [64] as follows: poor, κ < 0; light, κ = 0–0.20; fair, κ = 0.21–0.40; moderate, κ = 0.41–0.60; substantial, κ = 0.61–0.80; and almost perfect, κ = 0.81–1.00.

## Results

Of the 345 tested samples, 210 (60.9%) were infected with blood parasites after pooling results of ME and BCM testing (Additional file 1: Table S1). In BCM examination, *Haemoproteus* species were the most prevalent, followed by *Trypanosoma*, *Lankesterella*, microfilaria and *Leucocytozoon* parasites; no *Plasmodium* infections were detected by this method. In ME, *Haemoproteus* species were also the most prevalent, followed by *Plasmodium*, *Lankesterella*, *Leucocytozoon* and microfilaria parasites; no *Trypanosoma* infections were detected by this method (Table 1). Interestingly, BCM could detect approximately twice the number of co-infections than the applied protocol of ME. The most common co-infections detected by BCM were *Haemoproteus* and *Trypanosoma* parasites, while the most common co-infection was *Haemoproteus* and *Plasmodium* parasites when ME was used (Table 2).

**Table 1:** Prevalence of infection and sensitivity, specificity, positive predictive value (PPV) and negative predictive value (NPV) as well as Cohen’s Kappa index (Kappa) based on data obtained using buffy coat method (BCM) and microscopic examination (ME) of blood films

Parasite	Overall prevalence^a^	BCM^a^	ME^a^	Sensitivity^b^	Specificity^b^	PPV^b^	NPV^b^	Kappa^c^
*Haemoproteus*	135 (39.1)	125 (92.6)	113 (83.7)	91.2 (85.9–96.4)	90.5 (86.8–94.3)	82.4 (75.7–89.1)	95.5 (92.7–98.2)	0.80 (0.73–0.86)
*Trypanosoma*	91 (26.4)	91 (100)	0 (0)	–^d^	73.6 (69.0–78.3)	–^d^	100 (100)	–^d^
*Plasmodium*	18 (5.2)	0 (0)	18 (100)	–^d^	100 (100)	– ^d^	94.8 (92.4–97.1)	– ^d^
*Lankesterella*	13 (3.8)	10 (76.9)	7 (53.9)	57.1 (20.5–93.8)	98.0 (97.0–100)	40.0 (9.6–70.4)	99.1 (98.1–100)	0.46 (0.16–0.76)
*Leucocytozoon*	6 (1.7)	2 (33.3)	5 (83.3)	20.0 (0–55.1)	99.7 (99.1–100)	50.0 (0–100)	98.8 (97.7–100)	0.28 (0–0.72)
Microfilaria	6 (1.7)	6 (100)	1 (16.7)	100 (100)	98.6 (97.3–99.8)	16.7 (0–46.5)	100 (100)	0.28 (0–0.72)

^a^Number of positive samples followed by infection prevalence (in %) in parentheses

^b^Percentage, followed by the 95% confidence interval in parentheses

^c^Kappa index followed by the 95% confidence interval (in parentheses)

^d^Calculation not possible because the parasites were detected only by ME

*Note*: ME was considered as the gold standard method

**Table 2:** Prevalence of co-infections detected by buffy coat method (BCM) and microscopic examination (ME)

Co-infection	BCM^a^	ME^a^
*Haemoproteus* *+* *Lankesterella*	2 (0.6)	4 (1.2)
*Haemoproteus* + *Leucocytozoon*	1 (0.3)	1 (0.3)
*Haemoproteus* + microfilaria	2 (0.6)	1 (0.3)
*Haemoproteus* *+* *Plasmodium*	–	7 (2.0)
*Haemoproteus* + *Trypanosoma*	29 (8.4)	–
Microfilaria + *Trypanosoma*	1 (0.3)	–
*Plasmodium* + *Trypanosoma*	–	1 (0.3)
*Haemoproteus* *+* *Lankesterella* *+* *Trypanosoma*	4 (1.2)	–
*Haemoproteus* + *Plasmodium* *+* *Trypanosoma*	–	3 (0.9)
Total	39 (11.3)	21 (6.1)

^a^Number of positive samples, followed by infection prevalence (in %) in parentheses

*Haemoproteus* infections were observed in birds of almost all studied families, while *Lankesterella* parasites were more prevalent in birds of the Acrocephalidae, with majority of infections seen in the sedge warbler *Acrocephalus schoenobaenus.* Microfilariae and *Leucocytozoon* infections, were seen only in birds of the Accipitridae, Fringillidae, Corvidae, Muscicapidae, Phylloscopidae, Scolopacidae and Turdidae, while *Trypanosoma* species were more common and detected in 23 avian host species belonging to 11 families (Additional file 1: Table S1).

**Fig. 2:**
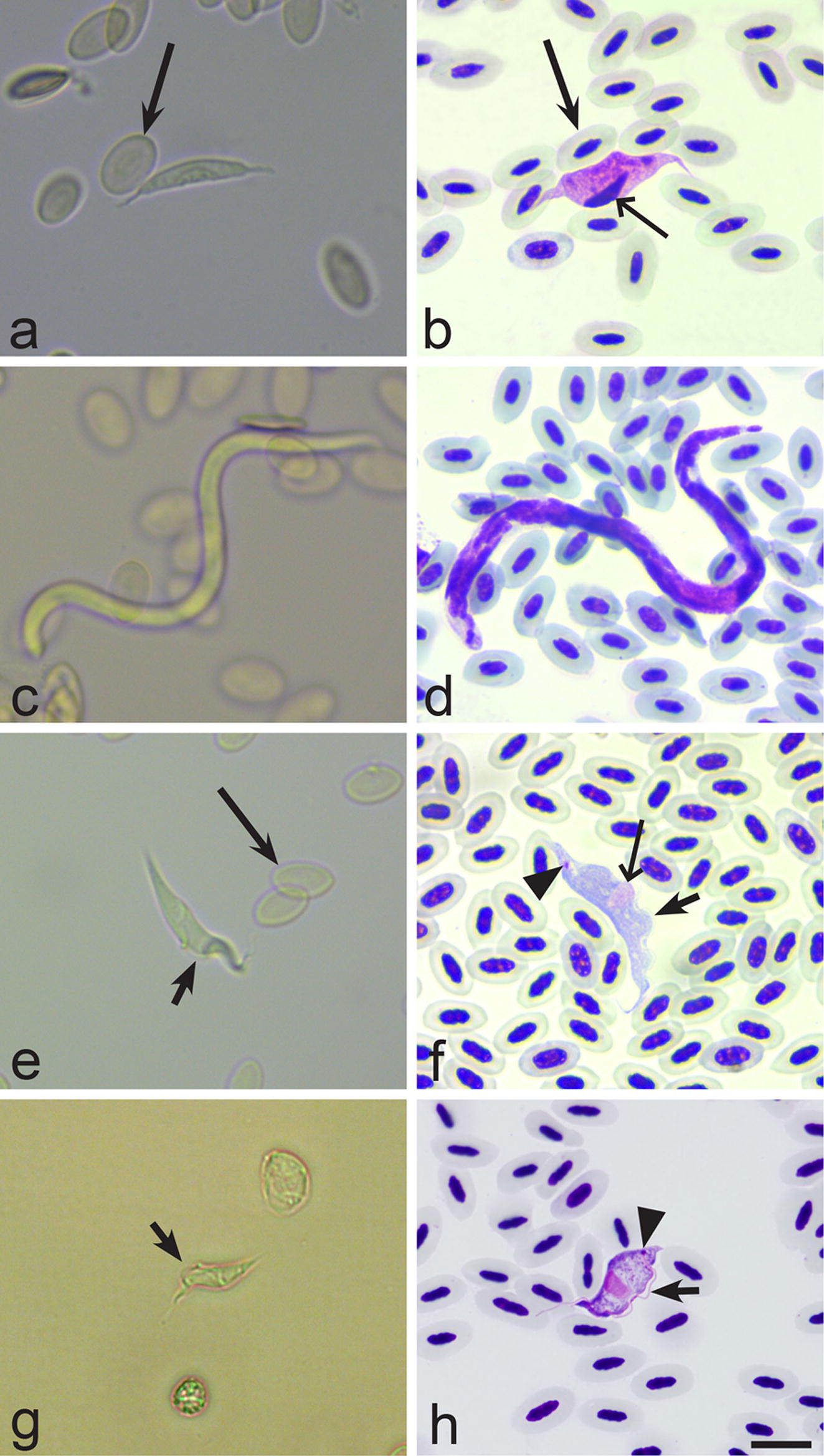
Images of relatively large blood parasites (larger than red blood cells) showing how they look in the buffy coat wet preparations (**a**, **c**, **e**, **g**) and methanol-fixed Giemsa-stained blood films (**b**, **d**, **f**, **h**). **a**, **b** *Leucocytozoon* sp. (barbed long arrow, red blood cell; simple long arrow, parasite nucleus). **c**, **d** Microfilaria. **e**, **f** *Trypanosoma* sp. (barbed long arrow, red blood cells; short arrow, undulating membrane; arrowhead, kinetoplast; simple long arrow, parasite nucleus). **g**, **h** *Trypanosoma everetti* (short arrow, undulating membrane; arrowhead, kinetoplast). *Scale-bar*: 10 µm

**Fig. 3:**
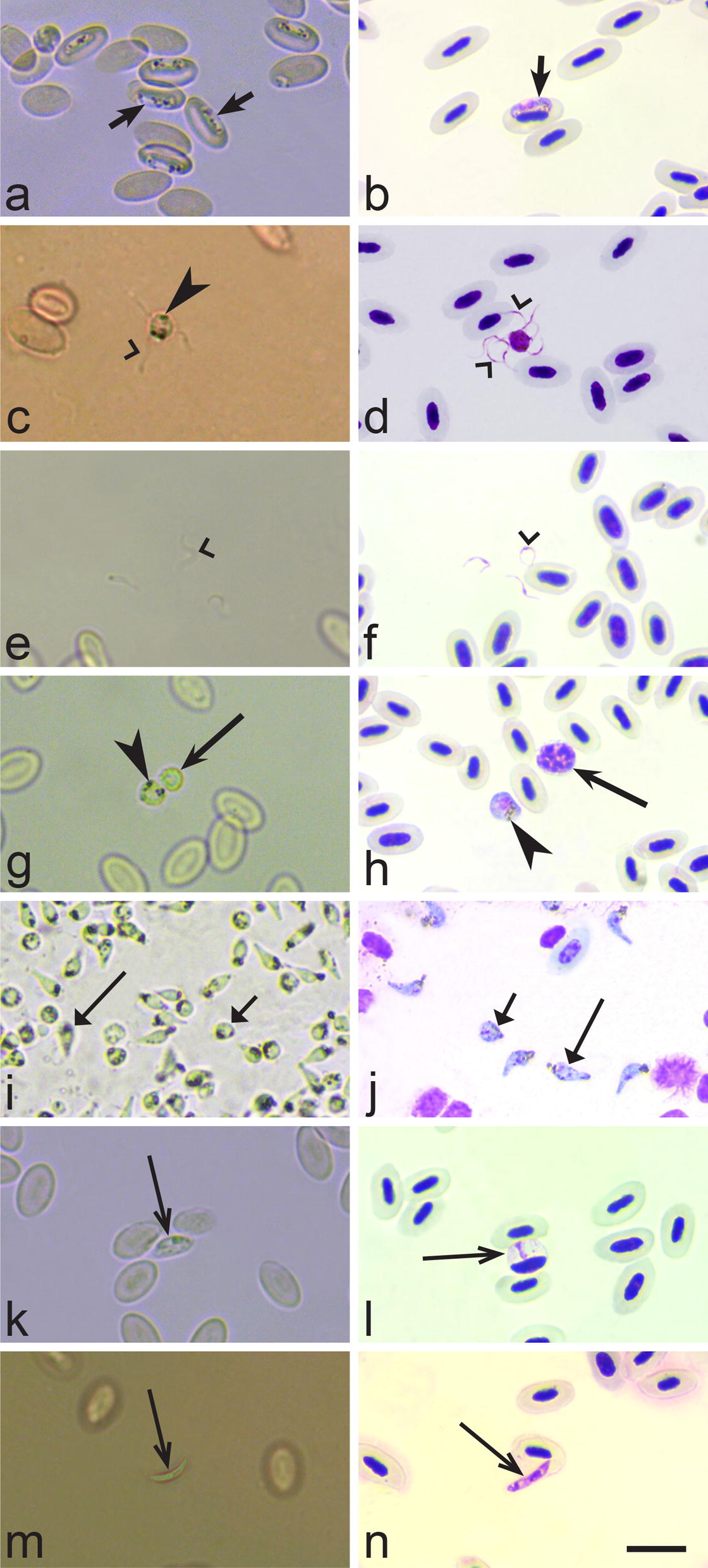
Images of relatively small blood parasites (smaller or equal to red blood cells in size) showing how they look in the buffy coat wet preparations (**a**, **c**, **e**, **g**, **i**, **k**, **m**) and methanol-fixed Giemsa-stained blood films (**b**, **d**, **f**, **h**, **j**, **l**, **n**). **a**, **b** Immature gametocytes of *Haemoproteus* sp. (arrows) inside red blood cells. **c**, **d** *Haemoproteus* sp. microgametocyte during exflagellation, note readily visible microgametes (simple wide arrowheads) still attached to extracellular microgametocytes (barbed arrowhead, pigment granules). **e**, **f** *Haemoproteus* sp. microgametes (simple wide arrowhead). **g**, **h** Extracellular rounded gametocytes of *Haemoproteus* sp. (left) and leucocytes (right, barbed long arrow); note that parasite can be readily distinguished from leucocytes due to presence of hemozoin pigment granules (barbed arrowhead). **i**, **j** Numerous *Haemoproteus* sp. ookinetes at different stages of maturation: immature (triangle wide short arrow), nearly mature and mature parasites (triangle wide long arrow) can be distinguished. Intracellular (**k**, **l**) and extracellular (**m**, **n**) *Lankesterella* sp. (simple long arrow); note that parasite is closely appressed to nuclei of mononuclear leucocyte (**k**, **l**). *Scale-bar*: 10 µm

During BCM examination, it was possible to observe parasites moving in the buffy coat wet preparations. Microfilariae were particularly easy to identify even before the buffy coat was transferred to the objective slide due to their large size and fast movement (Figs. 1b, 2c–d). *Trypanosoma* species were also easy to detect (Fig. 2e, f); however, due to their smaller size they were not so readily visible as microfilaria. The smallest trypomastigotes of the *Trypanosoma everetti* group are similar in length to red blood cells (Fig. 2g, h). It was possible to distinguish trypanosomes of this group because of their small size and an irregular shape, resembling a leaf or a kite in outline (Fig. 2h) rather than the usual spindle shape associated with trypanosome morphology [65]. *Leucocytozoon* infections were also detected using BCM, but mainly when relatively large fusiform host-parasite complexes were present (Fig. 2a, b). *Haemoproteus* spp. were readily observed, but as they are small parasites they were only seen after the buffy coat had been transferred onto objective slides (Fig. 3a, b). It is worth mentioning that mature gametocytes of *Haemoproteus* parasites start exflagellation a few minutes after exposure to air, and often it was possible to observe not only exflagellation (Fig. 3c, d), but also microgametes (Fig. 3e, f) and even fertilization events and ookinete formation (Fig. 3i, j). In regard to *Haemoproteus* identification using BCM, it is important to note that macrogametes of the parasites are of similar size as leucocytes, so it is crucial to pay attention to the presence of pigment granules, which are always present in blood stages of haemoproteids, in order to distinguish between these cells (Fig. 3g, h). Some species of *Leucocytozoon* were seen exflagellating as easy as *Haemoproteus* spp., but this process was rarely observed in leucocytozoids. *Lankesterella* infections were readily detected both as intracellular (Fig. 3k, l) and extracellular (Fig. 3m, n) parasites.

A comparison of the results of the ME and BCM diagnostics is shown in Table 1. Both methods were of a similar high sensitivity and specificity for the detection of *Haemoproteus* spp. (91.2% and 90.5%, respectively) and microfilariae (100% and 98.6%, respectively) infections. In regard to *Lankesterella* spp. and *Leucocytozoon* spp., BCM had a low sensitivity (57.1% and 20.0%, respectively) and high specificity (98.2% and 99.7%, respectively). Despite of the relatively high specificity for *Trypanosoma* spp. and *Plasmodium* spp. in BCM diagnostics, it was not possible to calculate the sensitivity of this tool because these parasites were only found using one method (Table 2).

Cohen’s kappa index (κ) showed substantial agreement (κ = 0.80) between BCM and ME (Table 1) for detection of *Haemoproteus* spp.; moderate agreement (κ = 0.46) for *Lankesterella* spp., and fair agreement (κ = 0.28) for microfilariae and *Leucocytozoon* spp. It is worth mentioning that although the agreement between these two diagnostic methods was substantial for *Haemoproteus* spp., the diagnostic results still did not agree with each other in 32 (9.3%) of the tested samples. In *Lankesterella* species diagnostics, which showed the lowest agreement between BCM and ME according to Cohen’s kappa index, the results differed in 8 (2.3%) of the tested samples.

## Discussion

The key result of this study is that the BCM is applicable for quick and reliable detection of avian blood parasites belonging to *Haemoproteus*, *Trypanosoma* and microfilariae, but is not recommended for *Leucocytozoon* and *Plasmodium* species diagnostics. This method could also be used for detection of *Lankesterella* infections; however, the sensitivity is low. This conclusion is important for fieldwork when infected donor birds should be rapidly screened and selected for parasitological and experimental research. This is a simple and quick parasite detection method, which can be used at each field site using simple microscopes and microcentrifuge equipment.

In addition to methodology results, this study contributes to the knowledge of the prevalence of *Haemoproteus*, *Plasmodium*, *Leucocytozoon*, *Lankesterella* and *Trypanosoma* species and microfilariae of filariid nematodes in common European birds (Additional file 1: Table S1). The obtained data corroborate with previous studies that reported prevalence of these blood parasites [66–69]. In the present study, *Haemoproteus* species had the highest prevalence (Additional file 1: Table S1). It is interesting to note that to date *Lankesterella* parasites have been reported only in the sedge warbler *Acrocephalus schoenoebaenus* (Acrocephalidae), Eurasian blue tit *Cyanistes caeruleus* (Paridae) and snow bunting *Plectrophenax nivalis* (Calcariidae) [3, 7, 70]. In the present study, the highest prevalence was reported in *Acrocephalus* birds, but *Lankesterella* infections were also seen in the lesser whitethroat *Sylvia curruca* (Sylviidae), European robin *Erythacus rubecula* (Muscicapidae) and northern wren *Troglodytes troglodytes* (Troglodytidae). It seems that *Lankesterella* parasites are more common in European birds than formerly believed; further investigation is needed on this matter.

The main difference between the BCM methods used for detection of blood parasites in humans and other vertebrates and the protocol developed in this study, is that the new protocol does not require any staining procedures, and the parasite diagnostic is possible using only the buffy coat wet preparations. The protocols used in human malariology [71–74] and *Trypanosoma* spp. diagnostics [46] apply capillary tubes containing acridine orange dye and require fluorescence microscopical examination for parasite visualization. In comparison to the BCM protocol described in the present study, methods which use fluorescence microscopy are more time consuming and expensive and also with limited application during fieldwork, particularly in remote areas. Additionally, we suggest examination of the buffy coat wet preparations, which is not the case in human malaria and *Trypanosoma* infection diagnostics. It is interesting that a protocol used in the diagnostics of microfilaria infections in dogs and humans [45] requires a mixture of blood, formalin and methylene blue. Although this protocol applies microscopic analysis of buffy coat wet preparations, all parasites are killed after such fixation.

BCM has a number of advantages in comparison to the gold standard ME. First, this tool does not require staining of blood film preparations thus, is easier to use during fieldwork. Secondly, the avoidance of fixation, drying and staining of blood films reduce all the variables that might result in poor quality of blood films [19]. Thirdly, BCM is faster than the gold standard ME. On average, BCM diagnostic results for one sample can be obtained in approximately 20 minutes after blood-sampling, while it takes about 40 minutes for the gold standard ME protocol, which was used in this study. If samples from ten birds are examined in parallel, BCM application saves over three hours on diagnostics, which is important during field studies. It should be mentioned that ME used in the present study is relatively fast in comparison to a precise microscopy protocol, which is normally used in blood parasite detection and is recommended in every-day laboratory research [2, 19] but is hardly applicable in the field as it is time-consuming (approximately 1.5 hours per sample). Fourthly, BCM gives good results for microfilariae and *Trypanosoma* species detection. The BCM has been known and extensively used in human medicine [45, 47, 48, 75, 76]. In veterinary medicine, it was mainly applied for examination of mammalian blood samples, with a focus on diagnostics of parasitic infections with zoonotic potential [47, 52, 53, 77–79]. This tool has been insufficiently applied in avian parasitology in wildlife [9, 58–61]. Fifthly, BCM can also be applied to distinguish between *Haemoproteus* parasites with fast and slow ookinete development since it is readily possible to see ookinetes being formed in the fast-developing species in the buffy coat wet capillary preparation (Fig. 3i). The feature of *Haemoproteus* parasites to extensively exflagellate quickly (within 10 minutes after infected blood is exposed to air) has been used in haemosporidian genomic research [40, 80] as well as gametogenesis and ookinete development studies [2, 81, 82], hybridization experiments [41] and vector studies [62, 83]. Sixthly, BCM not only leads to a concentration of parasites in one layer but it also uses a larger volume of blood (around 10 times more) than ME, which makes it more sensitive to detect a low parasitaemia [59]. Finally, BCM is more suitable to diagnose co-infections as using this method we were able to detect almost double the number of co-infections than ME (Table 2).

Although BCM is a promising technique for field research, it has some disadvantages. First, we recommend collecting about 30–50 µl of blood for BCM, which might be difficult in the case of small birds, for example the tiny passerine goldcrest *Regulus regulus* that is common in Europe. Secondly, non-moving parasites can be present both inside and out of the cells, as well as motile stages can occur and should be observed quite rapidly. As a result, some microscopy training might be needed before examination, since these forms are different from those that are seen on blood films stained with Giemsa. This is especially true for young forms of *Haemoproteus* spp. and *Plasmodium* spp. (Fig. 3a, b) that are not moving and would be found inside red blood cells as well as *Lankesterella* parasites (Fig. 3k–n) that move very slowly and are smaller than other blood parasites commonly found in birds. Thirdly, BCM is not recommended to be used for diagnostics of *Plasmodium* and *Leucocytozoon* infections. BCM fails to detect *Plasmodium* infections mainly because malaria parasites do not exflagellate when the blood is simply exposed to air, as is the case in all tested *Haemoproteus* species [40, 41]. *Haemoproteus* parasites develop readily visible moving stages (exflagellating microgametocytes and microgametes) in preparations prepared using BCM (Fig. 3d, f). Intracellular blood stages of malaria parasites are often small and difficult to visualize in non-stained preparations [2]. Although the leucocytozoids were seen in a few samples (Table 2), they are often at low intensity and might be difficult to distinguish from leucocytes. In BCM, *Leucocytozoon* parasites can be readily identified mainly when gametocytes develop in fusiform host cells. Such host-parasite complexes can be readily distinguished from all blood cells (Fig. 2a, b), but it is often difficult to distinguish between leucocytozoids and mononuclear leucocytes when the parasites develop in roundish infected host cells. It is worth noting that although exflagellation of some *Leucocytozoon* spp. has been described during simple exposure to air [2], this does not seem to happen readily for all *Leucocytozoon* parasites, as is the case in *Haemoproteus* species. Absence of pigment granules in *Leucocytozoon* spp. gametocytes markedly challenges visualization and identification of these parasites during application of the BCM. Fourthly, buffy coat wet capillary preparations can be stained for further microscopical analysis, but we do not recommend using this material to perform any morphological analysis, particularly parasite species description. The deformation of parasites after centrifugation was reported by Bennett [58] and we corroborate these findings. In *Haemoproteus* parasites, morphological changes might occur not only due to deformation during centrifugation, but also due to changes of mature gametocytes during sexual process and exflagellation that rapidly occur when the blood is exposed to air after withdrawal from birds (Fig. 3i, j). Another possible artefact, which might preclude microscopical examination in stained BCM preparations, might be due to the presence of high amounts of plasma, increasing the amount of protein and resulting in a dark pink staining of the background of the blood film. Despite these disadvantages, BCM is useful for the rapid detection of infected birds, which is essential for parasite detection and for further use in precise studies without harming the avian host.

When choosing a diagnostic method for parasite detection, it is important to known how sensitive and specific it is in comparison to other applied methods [63]. The present study shows that BCM can be recommended for use in diagnostic detection of *Haemoproteus* spp. and microfilariae infections, as the method has high sensitivity and specificity for both parasites (Table 2). In regard to *Lankesterella* infections, it is of relatively low sensitivity, but of high specificity (Table 2). However, BCM had the lowest sensitivity among encountered parasites for *Leucocytozoon* species despite of its high specificity. In regard to *Trypanosoma* and *Plasmodium* infections, we could not measure the BCM sensitivity because these parasites were only detected by one method, i.e. BCM or ME, respectively. These data suggest application of BCM as a diagnostic method for *Trypanosoma* infection and ME for *Plasmodium* infection, respectively.

It is interesting to note that the agreement between BCM and ME was considered as substantial in *Haemoproteus* infection diagnostics according to the Cohen’s kappa index (the number of positive samples detected by each of these methods coincided markedly) (Table 1). However, it is always desirable to reduce the time of sample screening during fieldwork, replacing the time-consuming techniques with less time-dependent methods, which certainly is the case with the BCM. The agreement between BCM and ME in detection of other parasites was lower than for *Haemoproteus* species; however, this study strongly indicates that the former method is markedly more sensitive in the diagnostics of *Trypanosoma* and microfilariae infections.

Results of diagnostics of human *Plasmodium* parasites using commercial kits containing capillary tubes with orange acridine were compared with data obtained using microscopical examination of Giemsa-stained thick blood films, which is considered the gold standard diagnose method for human malaria. Reported results were controversial; some studies showed a high sensitivity [74, 84, 85], while others reported a low sensitivity in the diagnostics [54, 86]. However, Adeoye & Nga [54] concluded that this is a useful protocol in early human malaria therapeutic intervention due to minimisation of diagnostic time. The present study corroborates this conclusion, particularly because BCM provides opportunities to analyse samples and select birds for experimental studies more quickly than during ME. Importantly, BCM can also be used for rapid blood parasite diagnostics in veterinary medicine, allowing treatment and/or prophylactic measures to start as soon as possible, with the aim to prevent further spread of disease.

## Conclusions

The quick detection of animals infected with parasites is often an important requirement in wildlife parasitology because it provides opportunities to minimize sampling time, resulting in less harm for individual animals and wildlife populations. That is particularly true in experimental research with avian blood parasites, which need to be selected from wildlife populations, in which infection prevalence is low and many host individuals need to be tested before the appropriate infected animals are selected. This study shows that BCM is a quick, reliable and powerful diagnostics tool, which is recommended to be used for detection of infections in birds, particularly of *Haemoproteus*, *Trypanosoma* and microfilariae parasites. This method is cheap, fast and simple to use, and thus is recommended for application during field studies even in remote areas. Importantly, this tool is sensitive for detection of blood parasite co-infections. It could also be applied in studies of blood parasites in other vertebrates, with the aim to increase the speed of diagnosis and to rapidly initiate animal treatment and application of prophylactic measures in disease control.

## Supplementary information

**Additional file 1: Table S1.** Prevalence of blood parasites reported in birds using the buffy coat method (BCM) and microscopic examination (ME) of blood films.

## Data Availability

All data generated or analysed during this study are included in this published article and its additional file.

## Abbreviations

BCM: buffy coat method
CI: confidence interval
κ: Cohen’s kappa index
ME: microscopical examination
PCR: polymerase chain reaction
