# Supplementary material for: The buffy coat method: a tool for detection of blood parasites without staining procedures
Source: Parasit Vectors. 2020 Feb 27;13:104. doi: 10.1186/s13071-020-3984-8 (PMC7045512; doi:10.1186/s13071-020-3984-8)
Supplement: Supplementary file 1 — Additional file 1: Table S1. Prevalence of blood parasites reported in birds using the buffy coat method (BCM) and microscopic examination (ME) of blood films. [file 13071_2020_3984_MOESM1_ESM.docx]

**Additional file 1. Table S1.** Prevalence of blood parasites reported in birds using buffy coat method (BCM) and microscopic examination (ME) of blood films.

| **Bird family and species** | **N** | **Number of birds infected with** | | | | | |
| --- | --- | --- | --- | --- | --- | --- | --- |
|  |  | **H** | **T** | **Pl** | **La** | **Mc** | **Le** |
|  |  | **BCM (ME)** | **BCM (ME)** | **BCM (ME)** | **BCM (ME)** | **BCM (ME)** | **BCM (ME)** |
| Accipitridae |  |  |  |  |  |  |  |
| *Accipiter nisus* | 1 | 1 (1) | 0 (0) | 0 (0) | 0 (0) | 0 (0) | 1 (0) |
| Total | 1 | 1 (1) | 0 (0) | 0 (0) | 0 (0) | 0 (0) | 1 (0) |
| Acrocephalidae |  |  |  |  |  |  |  |
| *Acrocephalus arundinaceus* | 5 | 2 (2) | 2 (0) | 0 (0) | 0 (0) | 0 (0) | 0 (0) |
| *A. palustris* | 3 | 2 (2) | 0 (0) | 0 (0) | 0 (0) | 0 (0) | 0 (0) |
| *A. schoenobaenus* | 26 | 19 (17) | 6 (0) | 0 (0) | 7 (5) | 0 (0) | 0 (0) |
| *A. scirpaceus* | 2 | 0 (0) | 0 (0) | 0 (0) | 1 (0) | 0 (0) | 0 (0) |
| *Hippolais icterina* | 1 | 1 (1) | 0 (0) | 0 (0) | 0 (0) | 0 (0) | 0 (0) |
| Total | 37 | 24 (22) | 8 (0) | 0 (0) | 8 (5) | 0 (0) | 0 (0) |
| Motacillidae |  |  |  |  |  |  |  |
| *Anthus trivialis* | 1 | 1 (0) | 0 (0) | 0 (0) | 0 (0) | 0 (0) | 0 (0) |
| *Motacilla alba* | 2 | 1 (1) | 0 (0) | 0 (0) | 0 (0) | 0 (0) | 0 (0) |
| *M. flava* | 4 | 1 (1) | 1 (0) | 0 (0) | 0 (0) | 0 (0) | 0 (0) |
| Total | 7 | 3 (2) | 1 (0) | 0 (0) | 0 (0) | 0 (0) | 0 (0) |
| Fringillidae |  |  |  |  |  |  |  |
| *Carduelis carduelis* | 7 | 1 (0) | 3 (0) | 0 (0) | 0 (0) | 0 (0) | 0 (0) |
| *Chloris chloris* | 2 | 0 (0) | 0 (0) | 0 (0) | 0 (0) | 0 (0) | 0 (0) |
| *Linnaria cannabina* | 12 | 2 (3) | 0 (0) | 0 (0) | 0 (0) | 0 (0) | 0 (0) |
| *Fringilla coelebs* | 14 | 11 (11) | 6 (0) | 0 (0) | 0 (0) | 1 (1) | 0 (0) |
| *Serinus serinus* | 2 | 0 (0) | 0 (0) | 0 (0) | 0 (0) | 0 (0) | 0 (0) |
| Total | 37 | 14 (14) | 9 (0) | 0 (0) | 0 (0) | 1 (1) | 0 (0) |
| Corvidae |  |  |  |  |  |  |  |
| *Corvus frugilegus* | 1 | 0 (0) | 0 (0) | 0 (0) | 0 (0) | 1 (0) | 0 (0) |
| Total | 1 | 0 (0) | 0 (0) | 0 (0) | 0 (0) | 1 (0) | 0 (0) |
| Hirundinidae |  |  |  |  |  |  |  |
| *Delichon urbicum* | 3 | 1 (1) | 3 (0) | 0 (0) | 0 (0) | 0 (0) | 0 (0) |
| *Hirundo rustica* | 18 | 2 (1) | 11 (0) | 0 (0) | 0 (0) | 0 (0) | 0 (0) |
| Total | 21 | 3 (2) | 14 (0) | 0 (0) | 0 (0) | 0 (0) | 0 (0) |
| Emberezidae |  |  |  |  |  |  |  |
| *Emberiza citrinella* | 5 | 1 (1) | 2 (0) | 0 (0) | 0 (0) | 0 (0) | 0 (0) |
| *E. schoeniclus* | 1 | 0 (0) | 0 (0) | 0 (0) | 0 (0) | 0 (0) | 0 (0) |
| Total | 6 | 1 (1) | 2 (0) | 0 (0) | 0 (0) | 0 (0) | 0 (0) |
| Muscicapidae |  |  |  |  |  |  |  |
| *Erithacus rubecula* | 5 | 1 (1) | 3 (0) | 0 (0) | 1 (0) | 0 (0) | 1 (2) |
| *Ficedula hypoleuca* | 5 | 3 (3) | 1 (0) | 0 (1) | 0 (0) | 0 (0) | 0 (0) |
| *Luscinia luscinia* | 2 | 1 (1) | 0 (0) | 0 (0) | 0 (0) | 0 (0) | 0 (0) |
| *Muscicapa striata* | 2 | 1 (1) | 1 (0) | 0 (0) | 0 (0) | 0 (0) | 0 (0) |
| *Phoenicurus ochruros* | 3 | 1 (1) | 2 (0) | 0 (0) | 0 (0) | 0 (0) | 0 (0) |
| *P. phoenicurus* | 7 | 1 (1) | 4 (0) | 0 (0) | 0 (0) | 0 (0) | 0 (0) |
| *Saxicola rubetra* | 4 | 1 (0) | 0 (0) | 0 (1) | 0 (0) | 1 (0) | 0 (0) |
| Total | 28 | 9 (8) | 11 (0) | 0 (0) | 1 (0) | 1 (0) | 1 (2) |
| Locustellidae |  |  |  |  |  |  |  |
| *Locustella naevia* | 2 | 0 (1) | 0 (0) | 0 (0) | 0 (0) | 0 (0) | 0 (0) |
| Total | 2 | 0 (1) | 0 (0) | 0 (0) | 0 (0) | 0 (0) | 0 (0) |
| Laniidae |  |  |  |  |  |  |  |
| *Lanius collurio* | 1 | 1 (1) | 0 (0) | 0 (0) | 0 (0) | 0 (0) | 0 (0) |
| Total | 1 | 1 (1) | 0 (0) | 0 (0) | 0 (0) | 0 (0) | 0 (0) |
| Alaudidae |  |  |  |  |  |  |  |
| *Lullula arborea* | 1 | 0 (0) | 0 (0) | 0 (0) | 0 (0) | 0 (0) | 0 (0) |
| Total | 1 | 0 (0) | 0 (0) | 0 (0) | 0 (0) | 0 (0) | 0 (0) |
| Passeridae |  |  |  |  |  |  |  |
| *Passer domesticus* | 1 | 0 (0) | 0 (0) | 0 (0) | 0 (0) | 0 (0) | 0 (0) |
| Total | 1 | 0 (0) | 0 (0) | 0 (0) | 0 (0) | 0 (0) | 0 (0) |
| Phylloscopidae |  |  |  |  |  |  |  |
| *Phylloscopus collybita* | 2 | 0 (0) | 1 (0) | 0 (0) | 0 (0) | 0 (0) | 0 (0) |
| *P. sibilatrix* | 4 | 1 (1) | 2 (0) | 0 (1) | 0 (0) | 0 (0) | 0 (1) |
| *P. trochilus* | 42 | 15 (13) | 14 (0) | 0 (0) | 0 (0) | 0 (0) | 0 (0) |
| Total | 48 | 16 (14) | 17 (0) | 0 (1) | 0 (0) | 0 (0) | 0 (1) |
| Sturnidae |  |  |  |  |  |  |  |
| *Sturnus vulgaris* | 18 | 8 (7) | 1 (0) | 0 (0) | 0 (0) | 0 (0) | 0 (0) |
| Total | 18 | 8 (7) | 1 (0) | 0 (0) | 0 (0) | 0 (0) | 0 (0) |
| Sylvidae |  |  |  |  |  |  |  |
| *Sylvia atricapilla* | 7 | 5 (5) | 3 (0) | 0 (0) | 0 (0) | 0 (0) | 0 (0) |
| *S. borin* | 2 | 0 (0) | 1 (0) | 0 (0) | 0 (0) | 0 (0) | 0 (0) |
| *S. communis* | 30 | 16 (16) | 4 (0) | 0 (6) | 0 (0) | 0 (0) | 0 (0) |
| *S. curruca* | 50 | 22 (17) | 16 (0) | 0 (10) | 1 (1) | 0 (0) | 0 (0) |
| Total | 89 | 43 (38) | 24 (0) | 0 (16) | 1 (1) | 0 (0) | 0 (0) |
| Scolopacidae |  |  |  |  |  |  |  |
| *Tringa glareola* | 43 | 0 (0) | 3 (0) | 0 (0) | 0 (0) | 2 (0) | 0 (0) |
| Total | 43 | 0 (0) | 3 (0) | 0 (0) | 0 (0) | 2 (0) | 0 (0) |
| Troglodytidae |  |  |  |  |  |  |  |
| *Troglodytes troglodytes* | 1 | 0 (0) | 0 (0) | 0 (0) | 0 (1) | 0 (0) | 0 (0) |
| Total | 1 | 0 (0) | 0 (0) | 0 (0) | 0 (1) | 0 (0) | 0 (0) |
| Turdidae |  |  |  |  |  |  |  |
| *Turdus merula* | 1 | 1 (1) | 1 (0) | 0 (0) | 0 (0) | 0 (0) | 0 (1) |
| *T. philomelos* | 2 | 1 (1) | 0 (0) | 0 (0) | 0 (0) | 1 (0) | 0 (1) |
| Total | 3 | 2 (2) | 1 (0) | 0 (0) | 0 (0) | 1 (0) | 0 (2) |
| **Grand total** | **345** | **125 (113)** | **91 (0)** | **0 (18)** | **10 (7)** | **6 (1)** | **2 (5)** |
| **Prevalence (%)** | **100** | **36.2 (32.8)** | **26.4 (0)** | **0 (5.2)** | **2.9 (2.0)** | **1.7 (0.3)** | **0.6 (1.4)** |

N: total number of sampled birds; H: *Haemoproteus*; T: *Trypanosoma*; Pl: *Plasmodium*; La: *Lankesterella*; Mc: microfilaria; Le: *Leucocytozoon*.
